# Exploration of Deaf People’s Health Information Sources and Techniques for Information Delivery in Cape Town: A Qualitative Study for the Design and Development of a Mobile Health App

**DOI:** 10.2196/humanfactors.6653

**Published:** 2016-11-11

**Authors:** Prangnat Chininthorn, Meryl Glaser, William David Tucker, Jan Carel Diehl

**Affiliations:** ^1^ Section of Design for Sustainability Department of Design Engineering Delft University of Technology Delft Netherlands; ^2^ Bridging Application and Network Gaps (BANG) Department of Computer Science University of the Western Cape Bellville South Africa; ^3^ Department of South African Sign Language School of Literature, Language and Media University of the Witwatersrand Johannesburg South Africa

**Keywords:** Deafness, sign language, South Africa, mHealth, health, qualitative research, co-creation, community-based co-design

## Abstract

**Background:**

Many cultural and linguistic Deaf people in South Africa face disparity when accessing health information because of social and language barriers. The number of certified South African Sign Language interpreters (SASLIs) is also insufficient to meet the demand of the Deaf population in the country. Our research team, in collaboration with the Deaf communities in Cape Town, devised a mobile health app called SignSupport to bridge the communication gaps in health care contexts. We consequently plan to extend our work with a Health Knowledge Transfer System (HKTS) to provide Deaf people with accessible, understandable, and accurate health information. We conducted an explorative study to prepare the groundwork for the design and development of the system.

**Objectives:**

To investigate the current modes of health information distributed to Deaf people in Cape Town, identify the health information sources Deaf people prefer and their reasons, and define effective techniques for delivering understandable information to generate the groundwork for the mobile health app development with and for Deaf people.

**Methods:**

A qualitative methodology using semistructured interviews with sensitizing tools was used in a community-based codesign setting. A total of 23 Deaf people and 10 health professionals participated in this study. Inductive and deductive coding was used for the analysis.

**Results:**

Deaf people currently have access to 4 modes of health information distribution through: Deaf and other relevant organizations, hearing health professionals, personal interactions, and the mass media. Their preferred and accessible sources are those delivering information in signed language and with communication techniques that match Deaf people’s communication needs. Accessible and accurate health information can be delivered to Deaf people by 3 effective techniques: using signed language including its dialects, through health drama with its combined techniques, and accompanying the information with pictures in combination with simple text descriptions.

**Conclusions:**

We can apply the knowledge gained from this exploration to build the groundwork of the mobile health information system. We see an opportunity to design an HKTS to assist the information delivery during the patient-health professional interactions in primary health care settings. Deaf people want to understand the information relevant to their diagnosed disease and its self-management. The 3 identified effective techniques will be applied to deliver health information through the mobile health app.

## Introduction

### Background

Deaf spelled with a capital “D” denotes membership of a cultural, linguistic minority group who choose signed language as their preferred language. This is as opposed to deaf with a small “d” that denotes someone with a hearing loss. Deaf people who mainly use signed language for communication experience disparity in information access in the majority hearing society [1]. Particularly in South Africa, Deaf individuals express the need to access understandable health information and communication to improve their well-being [2,3]. We have received similar messages from all the Deaf communities with whom we have been collaborating. Consequently, we took the initiative to design and develop a mobile health app called SignSupport and now wish to extend it with a Health Knowledge Transfer System (HKTS) [2,4]. This mobile app can support Deaf people’s communication at health facilities and can improve understanding of the diagnosed disease including self-management. Via a process of cocreation with Deaf communities and health professionals in Cape Town, we have gained an understanding to build the groundwork for the proposed HKTS. The app is meant to provide equitable information access as well as bridge communication gaps that are manifested by social barriers. An extended literature review led us to a number of social barriers that many Deaf people have faced since childhood.

### Social Barriers to Deaf People’s Access to Health Information

#### The Lack of Sign Language Within the Education of Deaf Learners

Signed languages cannot be translated word-for-word due to their structure distinct from spoken languages [5,6]. Driven by communication difficulties, social barriers are intrinsically formed. A standardized South African Sign Language (SASL) curriculum was not approved for teaching at schools for Deaf learners until 2012 [7]. As a result, many Deaf children in the past learned signed language from their peers [8]; which is how dialects developed and were passed through the generations across different regions of South Africa. Only 14% of their educators at schools for Deaf learners could use sign fluently which left many subjects untaught in SASL [9,10]. These educational barriers have resulted in average reading and writing skills of a Grade-4 level equivalent among Deaf school leavers [11]. Consequently, 75% of South African Deaf adults are functionally illiterate, and 70% of the Deaf population remains unemployed [12].

#### Disconnection From Hearing Family Members

Ninety percent of Deaf children are born to hearing families where many parents do not use signed language [13]. A Deaf child’s incidental learning of health information within the household usually fails due to language barriers. Health information, such as risks and dangers, from direct instructions by the parents or from “overhearing” conversations among family members cannot be understood by the Deaf child. Missing this kind of learning may have an impact on the physical and mental health, including the academic achievement of the Deaf person [14].

#### Noninclusive Health Information Through the Mass Media

Deaf people have very limited access to understandable health information available through the mass media, for example, newspapers, television, and the Internet. The majority of Deaf adults cannot understand jargon and technical terminology [15]. To a large extent, health information in the mass media is not presented in SASL, although some interpreting does appear on the news bulletins of South African TV channels. In addition, many Deaf people cannot afford Internet access to explore information, which is possibly available there in a signed language.

#### The Shortage of SASL Interpreters in the Health Care Context

There are no professional SASL interpreters (SASLIs) readily available at any health facility. Eighty-four SASLIs are currently registered at the Deaf Federation of South Africa to officially serve the Deaf population of around 600,000 [16,17]. The number of SASLIs who can interpret medical jargon is in even more critical shortage. In addition, the scarce SASLIs are too expensive for most Deaf people to hire for each health consultation [18]. The charge is between 250 and 350 South African Rand per hour excluding Value Added Tax; this may take up a 28% of the monthly Disability allowance of 1270 ZAR for a Deaf patient [19].

#### The Necessity of Providing Access to Health Information

##### Human Rights on Understandable Health Information

Everyone has the right to receive information with regard to a medical condition and in a language that she or he understands. The South African Health Act (61 of 2003) and Convention on the Rights of Persons with Disabilities 2006 both support the necessity of providing understandable health information to Deaf people. The first enforces, *“The health care provider concerned must, where possible, inform the user in a language that the user understands and in a manner which takes into account the user’s level of literacy* [20], ” and the latter states, *“The purpose of the present Convention is to promote, protect and ensure the full and equal enjoyment of all human rights and fundamental freedoms by all persons with disabilities, and to promote respect for their inherent dignity* [21].” Therefore, Deaf people are entitled to have access to health information in SASL, their own language, like all other patients.

##### To Induce Better Health

Many Deaf patients do not adhere to the suggested treatment or the prescribed medicines due to their limited health literacy as a consequence of poor access to understandable and accurate information. Some simply dispose of their prescribed medications if they do not understand the diagnosis or the importance of medication intake [3,22]. Others with chronic diseases purposely miss the follow-up visits by sending a hearing family member or a friend to get the repeat medication in order to avoid the confusing communication and inferior care [22,23]. Medical adherence would improve if the Deaf patients could understand their diagnosed condition and participate in the decision-making process for their treatment [24,25].

Therefore, together with our collaborators, we seek the opportunity to improve Deaf people’s access to health information and consequently their health through a mobile health app, SignSupport together with HKTS.

### Background About SignSupport and the HKTS

**Figure 1:**
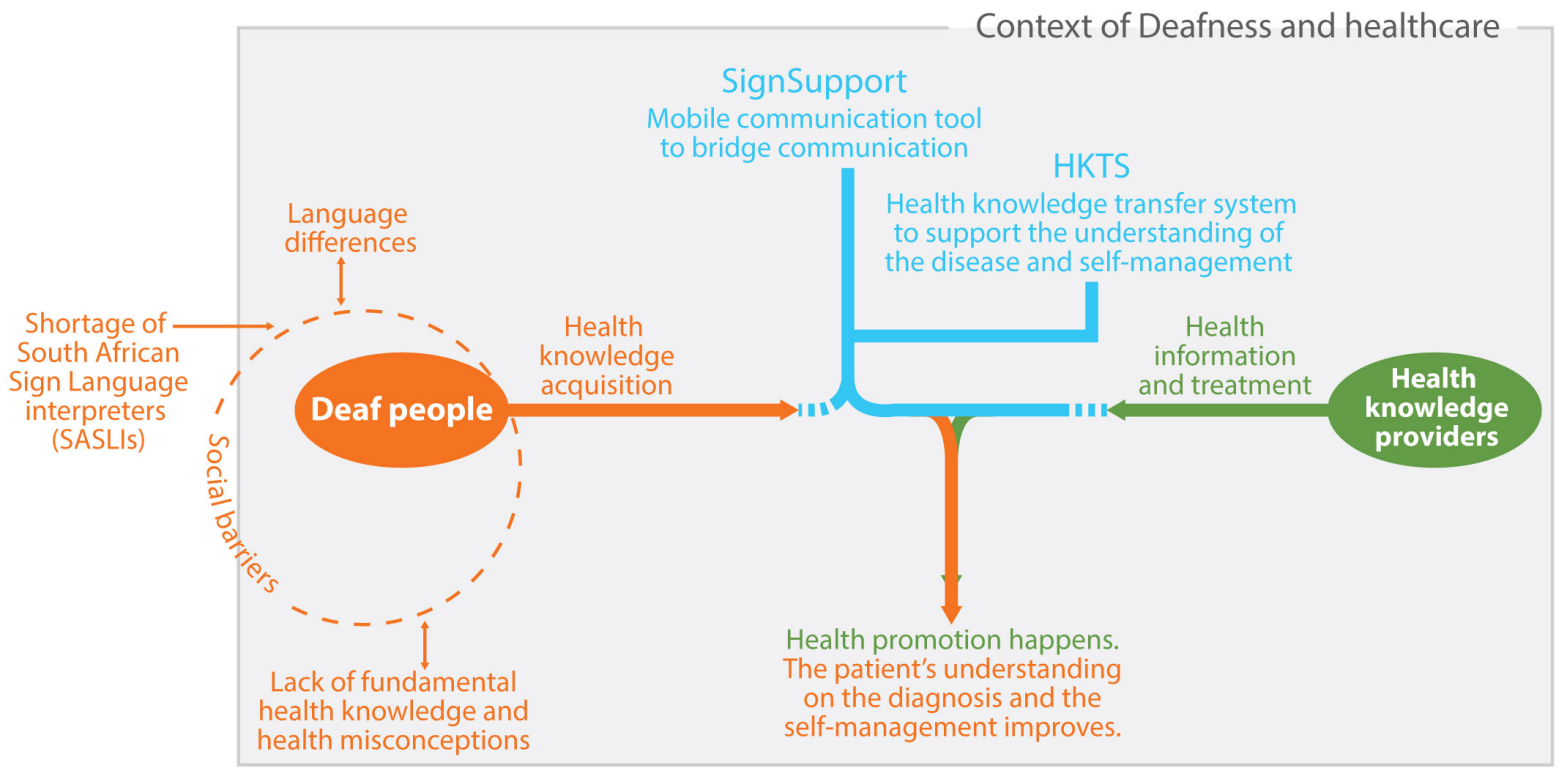
Overview of the design and development of SignSupport and Health Knowledge Transfer System (HKTS).

We initially started along this trajectory with a Deaf community in Cape Town. The theme “communication in a health care context” was prioritized to start the design and development of the mobile health app, SignSupport [2]. The research team later narrowed down the scope to focus on the medication dispensing process. This resulted in a SignSupport prototype which prompts a pharmacist to explain the prescribed medication instructions to a Deaf patient. The process of the explanation consists of making selections from provided options and taking photos of the medicine(s). The selections made by the pharmacist are matched with prerecorded SASL videos on the mobile device, which are then orchestrated as a set of medication instructions for the patient to view in SASL. From the usability test, Deaf participants reported their satisfaction with the use of SignSupport. Deaf participants could understand the medication instructions: medicine photo, dosage, medication intake time, recommendations, and warnings [26]. However, some of the Deaf participants revealed nonadherence to medication instructions. This was caused by health misconceptions shared within their community [27]. This is the point where the HKTS was conceived to provide Deaf users with understandable and accurate information of diseases and appropriate self-management (Figure 1), to provide more information to Deaf users beyond SignSupport, bridging the communication gap between the patient and the pharmacist.

Before writing this paper, the Deaf in Cape Town had confirmed mobile phones as their preferred tool for receiving and viewing health information. Within the same research session, many participants also suggested using diabetes as a case study for the design and development of the HKTS [4]. Our journey in building the groundwork for the HKTS was then given a specific context in which our mobile health app can be of use and the suitable techniques for delivering understandable health information to Deaf people. This paper therefore describes an exploration of which modes of health information delivery should be incorporated by the HKTS, and which effective presentation techniques can be applied.

### Related Work

#### Exploring the Information Sources Which People Use or May Use

Delivering health information at the right place and time can also increase the potential that the patient can improve their self-management [28,29]. Other projects that aim to develop accessible information sources for people with specific needs investigated on the information sources that people use and may use in the future. A consortium that was setting up an information center for the Deaf in Europe collected all the information sources Deaf people used. They learned the problems which Deaf people faced while using each information source in order to come up with possible solutions. Special needs retrieved from Deaf people were taken into consideration. Deaf people’s wishes on the future information center were also included in the study. All participants in the investigation wished for a pan-Europe information system with uniform standard for Deaf people in Europe [30]. Besides, understanding problems which the users of the information sources are facing, the trust issue should be as well investigated. Trust is an important component for one to take an action on the received health information [31,32].

#### Attempts to Distribute Health Information to Deaf People

There are a limited number of health information sources that provide health information in sign language. However, there are some websites that present health information in signed language, mainly in British Sign Language (BSL) or American Sign Language (ASL) for educational purposes. The following are examples of health information available via the Internet for Deaf people. Sign Health, developed by the Deaf Health Charity, supports BSL users with access to a large collection of videos related to health conditions and diseases. The information about each disease is signed by a BSL interpreter (BSLI), but no figures are used to accompany the explanations [33]. This information portal was originated after the report “Sick of It”—the report that shows the British Deaf people’s poorer health in comparison with their hearing counterparts [22]. The British Heart Foundation provides health information primarily for hearing people and some for Deaf people. The health information for Deaf people is explained using mixed techniques: combining motion graphics, narration by a BSLI, and sometimes subtitles [34]. Deaf Diabetes United Kingdom is a Deaf-led organization that provides support to Deaf people with diabetes. The informational materials on this website refer to the videos from the British Heart Foundation [35]. Deaf Health was developed to give clear and concise health information in ASL to the Deaf and Hard-of-Hearing community. The information available from this website is only narrated by ASL interpreters (ASLIs) [36]. Deaf Health by the University of California, San Diego (UCSD) provides information especially about different types of cancer. The information is presented by different combinations of techniques, for example, animation with simple and short text or subtitles and voice, and drama in signed language with subtitles and voice [37]. Noticeably, this accessible health information is mostly available for Deaf people in the rich economies, whereas it can hardly be found in other parts of the world. There is still no Web-based health information or mobile health information available for SASL users. As signed language is nonuniversal, this is an opportunity to explore the Deafness and health care context in South Africa for the design and development of the HKTS.

## Methods

### Approach

Through a community-based codesign (CBCD) approach, we involved both Deaf communities and health professionals in all phases of the action research (context exploration, planning, design and development, as well as testing and evaluation) in order to define suitable solutions toward the provision of equitable health information access to and for Deaf people [38,39]. We applied this qualitative research approach during the context exploration phase to answer the following research questions (RQs):

RQ1. What are the current modes of health information distribution available to Deaf people in Cape Town?

RQ2. What are the health information sources which Deaf people prefer and what are their reasons for this choice?

RQ3. What are the effective techniques to deliver understandable health information to Deaf people?

### Research Site and Participants

**Figure 2:**
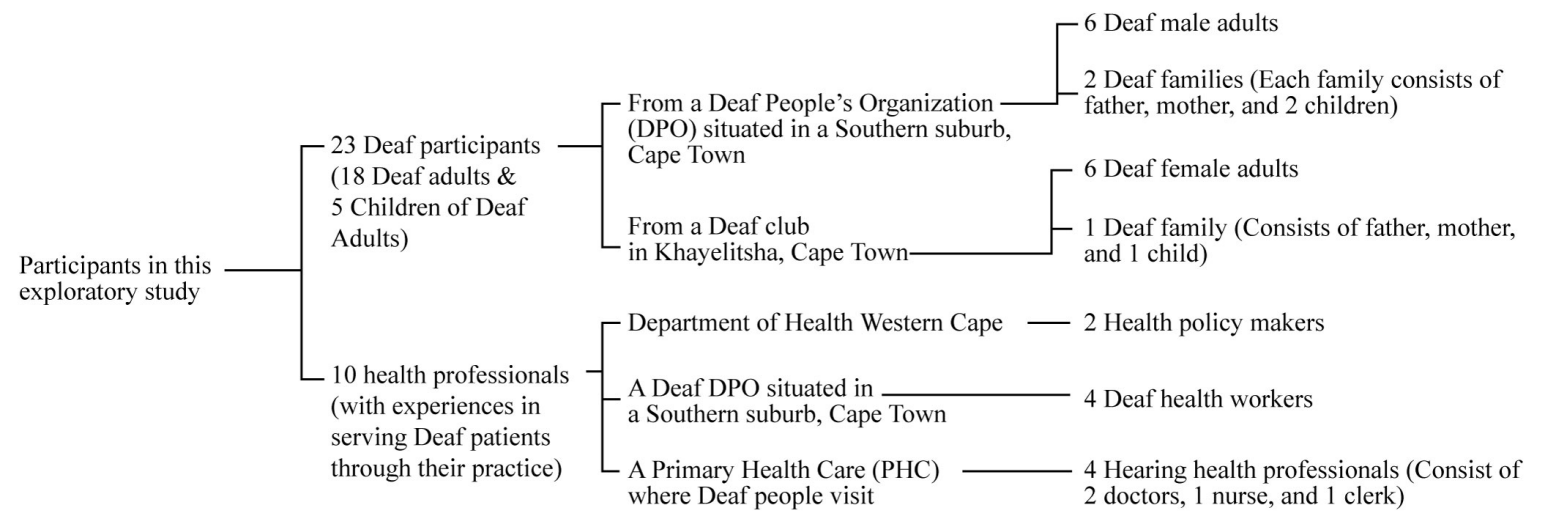
Participants in the exploratory study. DPO: Deaf People’s Organization.

The exploration took place in Cape Town during the period of January to May, 2014. A total of 23 Deaf participants and 10 health professionals were approached and invited to join interview sessions (Figure 2). It is important to note that these 10 health professionals were chosen because of their experience serving Deaf patients through their practice. In fact, these health professionals were specifically recommended by the Deaf communities with whom we worked.

### Procedure

A qualitative approach with a design-oriented methodology was applied for this exploratory study [40]. Two separate sets of semistructured questions were used for the interviews with groups of Deaf participants as the “information acquirers” and all health professional participants as the “health professionals.” Sensitizing tools were also used for retrieving extra insight information from the Deaf participants (Table 1). All Deaf participants were interviewed in groups. Based on our prior experience, Deaf participants tend to be more comfortable when they are among their peers; the discussion of nonprivate issues also becomes more dynamic. At the beginning of the interview, the participants agreed to allow each other an equal chance to give answers or share stories in response to the questions. The health professionals were interviewed either in a group or individually depending on their availability.

**Table 1:** Techniques used for data collection.

Participants		Techniques	Procedure run by a session facilitator
**Information acquirers**			
	Male group (Participants were not married nor had a child who could interpret for them)	Group interview: Semistructured questions with assistance from SASLI^c^	Step 1a: The research facilitator asked open-ended questions to explore the current health information sources that are available to Deaf participants. Then she wrote down each source that was mentioned on a sticky note. Step 2a: The research facilitator asked the Deaf participants to share their experiences and techniques used during receiving or acquiring health information from the abovementioned sources. Step 3a: (Only with the Deaf families groups) The research facilitator asked the participants to explain if their hearing CODAs are considered as their health information source and if they have any informational influence on them as parents. Step 4a: The research facilitator showed the evaluation map and gave the written sticky notes to the participants. Then she asked the participants to discuss within the group the accessibility of each mentioned information source with reference to their access to this source, the techniques used for information delivery, and the comprehensibility of the retrieved information. The sticky notes are then placed in the areas of degrees of accessibility they agree on, and they reflect on their reasons. At this step, we derived *“the list of the current health information sources”* that Deaf people can access. Step 5a: The research facilitator asked the participants to discuss within the group the information sources they wish to have available to them. Then they wrote down each source they wish to have available on a sticky note. This resulted in *“the wished-for sources.”* Step 6a: The research facilitator asked the participants to discuss and adjust the positions of all sticky notes (with the current health information sources and the wished-for health information sources) on the evaluation map of accessibility. Then she asked them to reflect on the reasons for these decisions. From this step, we derived *“the extended list with the wished-for sources”* added.
		Sensitizing tools: - Sticky notes with Deaf participants’ mentioned health information sources - Evaluation map of the accessibility of the mentioned information sources (5 areas on the map indicate the degrees of accessibility, from the highest to the lowest)	
	Female group (Participants were not married nor had a child who could interpret for them)		
	Deaf families consisted of Deaf parents and hearing children (the so-called CODA^a^)		
**Health professionals**			
	Health policy makers	Group interview: Semistructured questions with assistance from SASLI	Step 1b: The research facilitator asked open-ended questions to understand the responsibilities in terms of health information distribution to all the patients. Step 2b: The research facilitator asked the participants to share their experiences and the techniques used in delivering health information to Deaf patients.
	Deaf health workers		
	Hearing health professionals at the PHC^b^ facility	Group interview or individual interview: Semistructured questions	

^a^CODA: child of Deaf adult.

^b^PHC: primary health care.

^c^SASLI: South African Sign Language interpreter

### Data Analysis

All interviews were recorded on video and audio formats. Both inductive and deductive coding was applied to the analysis. The indepth information retrieved from different groups of Deaf participants was combined in order to define the modes of health information distribution to Deaf people and their preferred health information sources. The information retrieved from Deaf participants and health professional participants was later used to verify the health information delivery techniques that were found to be effective or ineffective.

### Ethical Considerations

We received ethics approval from the Health Research Ethic Committee of Delft University of Technology and from the Institutional Research Board of the University of the Western Cape for this research. The research purpose, risks, and benefits of the design and development of the HKTS, rights of participants, and identity protection were communicated to all participants in advance of any interview. Certified SASLIs, who are also accepted by the participating Deaf communities, assisted to relay the communication with all Deaf participants. The informed consent from the Deaf participants was recorded via raised hands in front of a video camera. We addressed many, if not all, of the ethical concerns that arise when dealing with Deaf participants [41].

## Results

### Current Modes of Health Information Distribution

**Figure 3:**
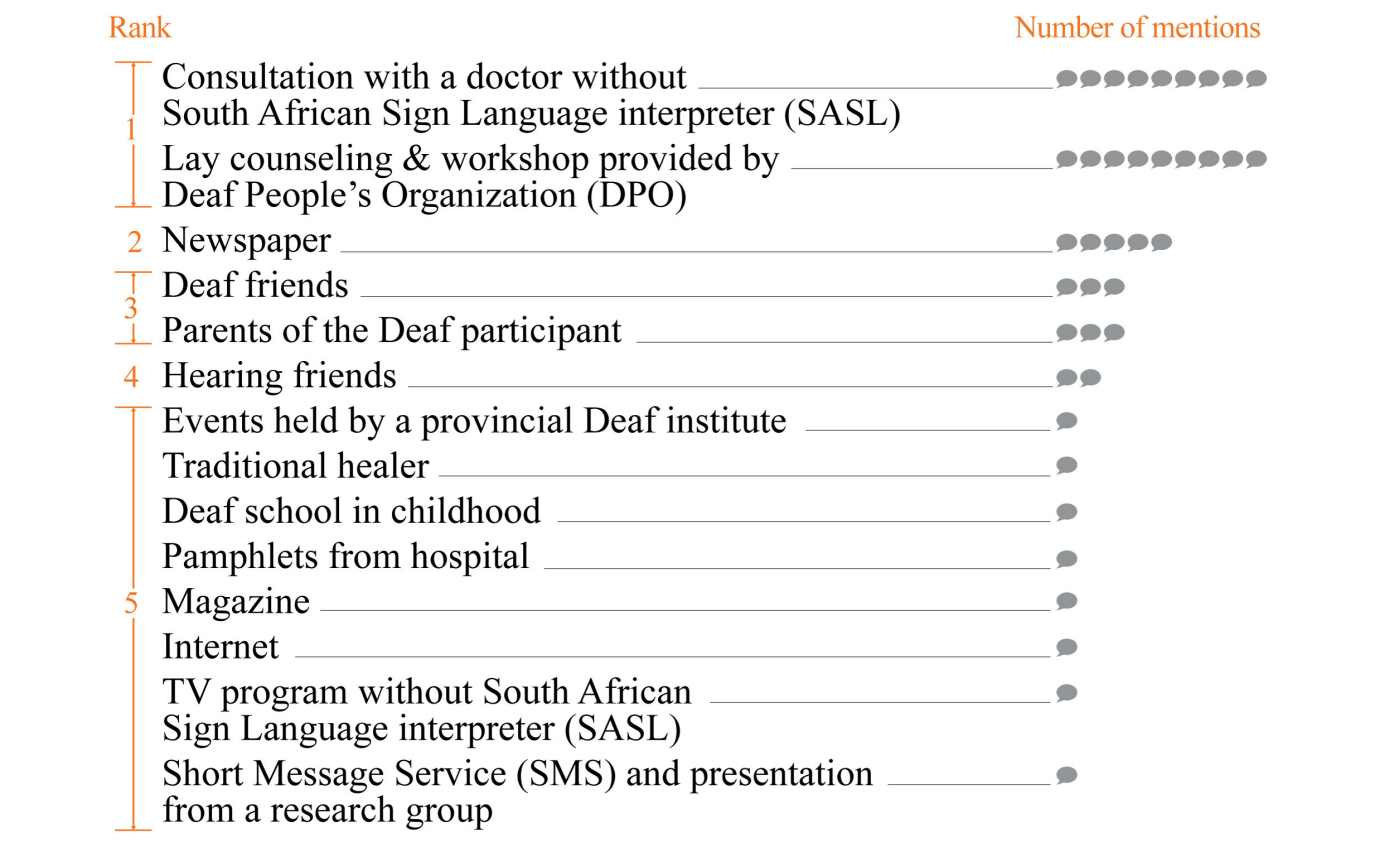
The current health information sources available to Deaf people.

The exploration shows that under the limitation, Deaf people approach some sources to get health information from. The Department of Health (DoH) in the Western Cape sets a health calendar for national and international health days each year. The DoH distributes the mandated health information to the Deaf population through Deaf and other relevant organizations. Deaf people also have the opportunity to receive health information from consultations with health professionals or from the mass media despite the aforementioned limitations. In addition, they randomly receive information through personal interactions with their Deaf peers and a few hearing friends or family members. Figure 3 illustrates 14 information sources, which the information-acquirers mentioned as being available to them. The ranking was composed according to the amount of times each source was mentioned.

The 14 sources were then coded into themes and clustered into 4 modes of health information distribution for answering RQ1. The details including feedback from participants on each of the 4 modes are as follows:

#### Health Workshops and Counseling Offered by Deaf and Other Relevant Organizations

##### Workshops and Lay Counseling Offered by Deaf People’s Organizations

In 2014, there were 5 health workers, who are also Deaf, across Cape Town. All of them were located at one Deaf People’s Organization (DPO). The Deaf health workers were mainly trained for HIV/AIDS lay counseling [42]. They currently collaborate with other relevant organizations to promote health information to Deaf members according to the DoH’s health calendar. The health workers and the auxiliary members presented information using 2 communication strategies: (1) private and confidential counseling for individual clients with HIV/AIDS and (2) workshops and dramas in SASL for a mass signing audience. The lay counseling aims to identify HIV/AIDS-infected members for timely assistance in self-management and treatment-adherence education. The health workers performed social and health dramas in SASL for Deaf members during their monthly gatherings and also with outreach programs around the Western Cape to smaller Deaf communities. The dramas cover common and relevant health misconceptions gathered through their casework. A short presentation with pictures is subsequently presented to the audience. The session ends with an open platform for questions and answers.

Deaf participants like the health dramas because they are in SASL. As a result, the story and the arguments are easy to follow:

When there is drama, you get to understand something you never understood, so it’s very good.

In addition, a few Deaf participants wished to review the dramas at their own time and place of convenience due to their limited budget for traveling from home to the DPO.

The Deaf health workers also find the combined techniques effective in delivering and simplifying health information. They compose the drama to imitate the daily lives of Deaf people, so it helps Deaf people let go of common misconceptions. The short presentation is used to further explain the topic; and pictures are used to enhance the audience’s understanding during the presentation. At the end of the session, the open platform for questions and answers provides opportunities for the audience to clarify their doubts based on the characters in the drama without revealing their personal problems.

##### Events Held by a Provincial Deaf Institute

The information is also presented to the Deaf audience in SASL through the assistance of the certified SASLIs.

##### Health Education From School

Health education given during one’s schooling is considered by a Deaf participant as the information source that lays down some fundamental health knowledge for the person.

##### Health Texting and Presentations From a Research Group in Cape Town

A text message (Short Message Service, SMS) is written in simple English, isiXhosa, or Afrikaans, which Deaf people can understand. Although some participants from our Deaf-female group mentioned that only a few of them understood the SMS text messages, they all agreed that it was still better than receiving nothing. In addition, since 2008, this research group has been offering the first free-of-charge SASL interpreting to Deaf outpatients with advance booking prior to a health facility visit [43].

#### Consultations With Hearing Health Professionals

As with all health professionals, the ones participating in this study have their own roles to play in their aims and responsibilities to maintain wellness, prevent illness, and promote health during face-to-face interaction with all patients. Group communication forums for chronic diseases, small support groups for HIV, and health education in the waiting areas at the health facilities are additional communication strategies that PHC system uses for optimizing health promotion to specific groups of patients. Given the situation that most if not all health professionals are hearing, and in this case, dealing with Deaf patients, the health professionals must also address the need for assistance from SASLIs at health facilities.

All Deaf participants emphasized the communication problems they experienced at the health facilities in the absence of an SASLI. Deaf patients who had no SASLI as an escort had to communicate via writing or lip-reading, which is not preferred. This led to confusion and frustration for the patient when one could not understand his or her diagnosis. Several Deaf participants admitted that their nodding during the consultation was to rush the consultation to an end; it did not refer to their understanding:

When it comes to writing back and forth with the doctor, it’s difficult to deal with. You will say (nod) yes, yes, yes to everything. And then when you go outside, you will ask people what it means because when you stop them (doctors), they get furious.

Deaf participants from Deaf family groups who sometimes had an SASLI or a CODA escorted them to a repeat appointment for a chronic disease, in contrary, had better experiences during the consultations. They understood the test results, the treatment planning, and medication adjustment:

When the interpreter is there, she will communicate with the doctor and then will sign to me. I understand everything perfectly. The same applies when I go to the pharmacy, when the interpreter is there, it’s easy to explain how to use medication, and if your blood pressure is high or your diabetes is high. So it is easy when the interpreter is there. But when the interpreter is not there, there can be some misunderstanding on medication and others things, so I always go with a sign interpreter when going to a public hospital.

Many health professionals routinely wrote or merely shouted while communicating with their Deaf patients since they did not understand Deaf people’s backgrounds and their specific communication needs. Some health professionals who understand a little SASL would avoid signing as it could cause miscommunications. Drawing may be used to explain the time for medication intake. A doctor from the interviews demonstrated his explanation of a disease progression to the Deaf patient in analogy, whereas another doctor would rather explain only the important actions that the patient must take to avoid further confusion. Therefore, a Deaf patient usually does not receive complete information about the diagnosed disease, treatment planning and its options, self-management, and schedules for follow-up appointments. Due to the communication gaps, the health professionals could not check-back their patient’s understanding of the explained subject.

The health policy makers are aware of these communication problems among Deaf patients and health professionals and the shortage of SASLIs in the health care context. They additionally understand that the support groups provided for hearing patients are not suitable for Deaf patients. Therefore, they are still looking for solutions that optimize the use of existing information and communication technologies to distribute inclusive health information for all.

#### Information Shared Through Personal Interactions

##### Deaf Friends

Deaf friends who can read become the immediate information source to others. These friends can give simple advice, read and explain medication instructions, or suggest one to a health facility with Deaf-friendly staff. On other hand, several health misconceptions are commonly shared through the close-knit relationships in the Deaf communities [15].

##### Hearing Friends

A participant mentioned partial health information received from hearing friends; however, another participant additionally revealed a miscommunication received from his hearing friend about smoking and health. Both participants showed a similar pattern of language barriers as a problem while communicating with their hearing friends.

##### Parents of the Deaf Person

Three participants received some advice from their mothers concerning their own or their partner’s pregnancy. On the opposite side, none of the Deaf participants considered their CODAs as their health information source, although the children occasionally shared some information with them, for example, lifestyle modification for better health.

#### Mass Media

##### Printed Media

Five participants read the newspaper and found some interesting health information although they did not always understand the terminology used in the articles. One participant who experienced problems while communicating with a support group prefers self-study via pamphlets with pictures distributed at the health facility *.* One other participant likes reading information concerning her chronic disease from her favorite magazine. These participants construct their understanding from the wording they understand in combination with the accompanying pictures, although they could not understand all the terminology used in the content.

##### TV Programs

A participant followed her favorite program that presented health-related information. She used her lip-reading skills in combination with the visual graphics that appeared on screen to construct her understanding. She might also ask her CODA to relay the information.

##### Internet Browsing

A participant frequently browsed the Internet to acquire further information about the terminology found elsewhere. However, most of our Deaf participants do not have access to the Internet or adequate computer literacy skills.

### Preferences of Deaf People on the Health Information Sources

**Figure 4:**
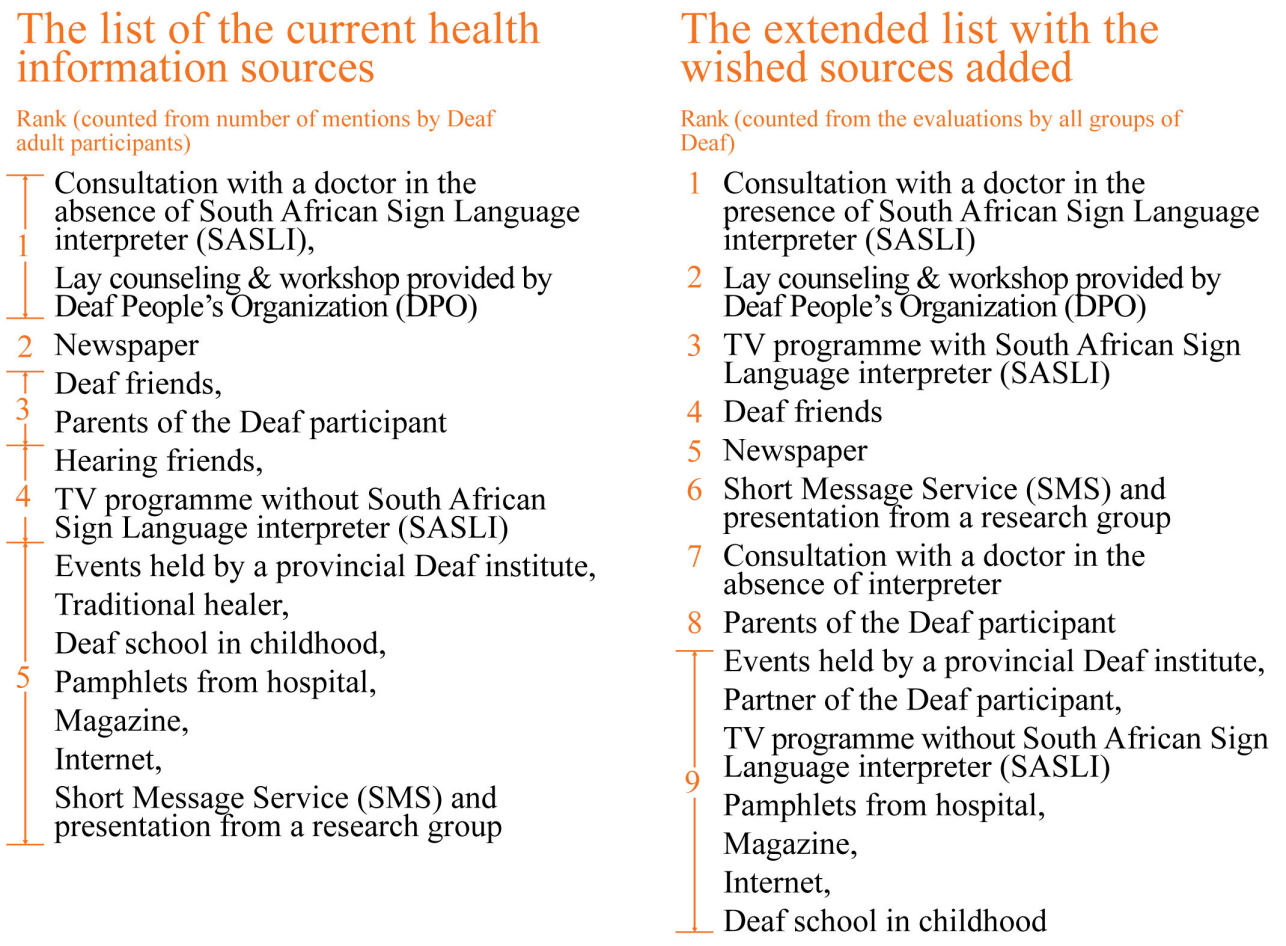
Comparison of the list of the current health information sources and the extended list.

The participants were asked to discuss health information sources that they wish to be available for Deaf people (Table 1: Step 5a). The wished-for sources were added to the list of current information sources. The participants subsequently evaluated the extended list of health information sources on comprehensibility with a focus on language and communication techniques used. This list contained the preferred health information sources which comprises the answers to RQ2. This evaluation resulted in a new ranking which reflects the preferences among Deaf people for accessible health information sources. By comparing the 2 lists (Figure 4), we noticed that Deaf participants wished to have SASLIs for most services available publicly. Having an SASLI available at health facilities is the most desired situation in this context because they need to understand their health conditions at the time of seeing the health professional. Having the counseling and workshop provided by the DPO and SASL interpreting on TV for health information also increases the opportunities during which Deaf people can learn to take care of their health.

### Techniques of Delivering Health Information

From all the participant’s feedback, 3 effective techniques for delivering understandable health information to Deaf people were defined. These are the answers to RQ3. In addition, 2 ineffective techniques are additionally described for acknowledgment. These techniques are presented in no particular order.

#### Effective Techniques

##### Information Delivery in SASL

The responses from Deaf participants, Deaf health workers, hearing health professionals, and policy makers confirmed that delivering health information in SASL is the most important element for Deaf patients. Efficient methods of delivering information in different dialects should also be considered.

##### Health Dramas With Combined Techniques

Complicated subjects or topics can be simplified and made memorable through SASL drama for a Deaf audience. The Deaf health workers usually combine this effective technique with a short presentation and an open platform for questions and answers. These combined techniques helped Deaf people to confront the facts and undo the health misconceptions, which they had held for a long time.

##### Pictures in Combination With Simple Text Descriptions

We learned that pictures in combination with simple text descriptions can help Deaf patients construct and enhance their understanding about the information. The descriptions could be in English or any other written language which the Deaf patients are familiar with. This finding corresponds to the findings that the scientific principles or processes must be made visual for Deaf learners in order to be understood [44]. As we noted in the introduction, many Deaf people are functionally illiterate with written language, in this case English, Afrikaans, or isiXhosa. However, evidence has shown that even with limited textual capabilities, Deaf people regardless frequently use text to communicate with each other via SMS [45] and undoubtedly now with apps like WhatsApp, Facebook, and email; and further, Deaf people do wish to learn textual literacy as evidenced by the long-running English literacy project at the Deaf Community of Cape Town, the use of the text at tertiary level at the National Institute of the Deaf, for example, and others. Having both SASL video and text side-by-side in an app could potentially offer benefits in this regard. In addition, it must be noted that for the sake of the developers, having textual “bread crumbs” in the human computer interface greatly assists with keeping track of content (although it must be matched up rigorously with SASL video content) [26].

#### Ineffective Techniques

##### Functional Literacy Requiring Information

Writing is an ineffectual technique to deliver health information to Deaf patients because many Deaf people are less skilled in reading and writing. Heavy text content with jargon and complicated terminology will lose their attention. Similar findings were made by other studies related to health information delivery to Deaf people in different countries [46-48].

##### Lip-Reading Skills Requiring Information

Lip-reading is not preferred by Deaf people. The accuracy of English lip-reading is only 30-35% [49]. In addition, no patient could read the doctors’ lips while they are wearing a mask. Unfortunately, many health professionals do not realize this issue because they have limited understanding of Deaf people’s communication requirements [2].

## Discussion

### Principal Findings

From this exploratory study, we found 4 modes of health information distribution that are currently available for Deaf people in Cape Town. Based on these modes, we also gained an understanding of Deaf people’s preferred health information sources. The Deaf people based their preferences of the information accessibility on the language and the communication techniques used by each information source. The effective techniques for delivering the understandable health information to the Deaf users will be applied to the design and development of the HKTS. Delivering health information in SASL will significantly provide increased accessibility to Deaf people, especially on a low-cost mobile device. The video drama, combined with other techniques, is seen as a particularly innovative way to present and simplify health information to a Deaf audience. The use of pictures in combination with simple text descriptions can provide opportunities for Deaf people with low functional literacy to construct an understanding of the explained subject, recalling that Deaf people with much stronger sign language literacy yet are still interested to acquire textual literacy as it is by necessity needed to integrate into the greater hearing world.

While building the groundwork for the design and development of the HKTS, we learned that the mobile phone is the preferred communication tool for Deaf people to receive and view the health information. The Deaf communities also suggested diabetes care as a subject for the HKTS. From this exploratory study, we have defined 3 effective techniques for delivering understandable and accurate health information which Deaf people need. In addition, we see the opportunity for the HKTS to assist the health professionals in delivering understandable information to a Deaf patient, especially when an SASLI is absent. Deaf people consider that timely understanding of their health condition during consultation is very important. We will focus on the communication between a Deaf patient and health care staff at PHCs as the problem in delivering health information prominently occurred in that specific setting. The next phase of the research will be to cocreate the HKTS among the Deaf people, health professionals, and the research team. The content-specific health information within the HKTS will be determined to meet both parties’ requirements. Inputs from all participants are valuable to help us verify the attributes of the systems.

### Limitations

The Deaf participants who we invited from 2 Deaf communities appear to have connection and access to similar health information sources. It is possible that Deaf members of other Deaf communities in Cape Town, who we did not invite to participate in the focus groups, may have access to different health information sources. This may also result in different preferences on the sources. Their responses that were not collected may also lead to different effective techniques in delivering understandable and accurate health information. In addition, we need to take into account the different needs among Deaf communities when applying our findings to other Deaf communities outside Cape Town.

We realize the probable but unavoidable (inter)subjectivity and therefore the bias which influences the analysis of the results from this purely qualitative study. Of course our results might not be replicable as they target specific communities with low sample sizes. However, it is also accepted that qualitative methods such as ethnographic action research [50] and community-based codesign [51] can yield results that are transferable, for example, from one community to another. Furthermore, we also designed the responses in this study redundantly to assist in triangulating toward transferable results: the participants give their answers (Table 1: Step 1a and 1b), reflect their reasons (Table 1: Step 2a, 3a, and 2b), and affirm their answers (Table 1: Step 4a, 5a, and 6a). This is to extract the “real” meaning of the answers given by the participants as valid as possible, and likewise reducing the bias by the researcher during the data analysis. For the purpose of this study we can accept these limitations, as we toil in the action research space, in our case with various small Deaf communities. In other words, we aim for transferability over generalizability [52], and claim that our results and recommendations are as valid as quantitative methods; only that in our case, qualitative methods are better able to address the chosen research problems **.**

### Conclusions

With regard to RQ1 (What are the current modes of health information distribution available to Deaf people in Cape Town?), Deaf participants mentioned 14 health information sources that they can access. The sources can be clustered into 4 modes of health information distributed to Deaf people in Cape Town, viz, (1) health workshops and counseling offered by Deaf and other relevant organizations, (2) consultations with hearing health professionals, (3) information shared through personal interactions, and (4) the mass media.

With regard to RQ2 (What are the health information sources which Deaf people prefer and what are their reasons for this choice?), Deaf people base their preferences, whether an information source is accessible, on 2 factors viz, (1) that it delivers information in signed language; and (2) that it uses techniques to simplify the topic and to help Deaf people construct their understanding. These factors make the consultation with a doctor in the presence of an SASLI, lay counseling and workshops provided by a DPO, and TV programs with SASLI rank as the top 3 of the extended list in Figure 4.

At the end of the analysis, with regard to RQ3 (what are the effective techniques to deliver understandable health information to Deaf people?), we found that there are 3 effective techniques to deliver understandable health information to Deaf people. The information delivery in SASL including its dialects is the most important element of the accessible information because it is the language that Deaf people mainly use for communication in Cape Town, South Africa. The health drama with combined techniques, as optimized by a DPO, helps in simplifying complicated topics; followed by a short presentation and an open platform for questions and answers helps Deaf people to debunk the health misconceptions they may have. Pictures in combination with simple text descriptions accompanying the health information helps the Deaf information-acquirers construct and enhance their understanding on the explained subject. These effective techniques will be applied for the future design and development of the HKTS.

## Abbreviations

SASL: South African Sign Language
SASLI: South African Sign Language interpreter
BSL: British Sign Language
BSLI: British Sign Language interpreter
ASL: American Sign Language
ASLI: American Sign Language interpreter
UCSD: University of California, San Diego
HKTS: Health Knowledge Transfer System
CBCD: Community-based codesign
RQ: Research question
PHC: Primary Health Care
DoH: Department of Health
DPO: Deaf People’s Organization
SMS: Short Message Service
